# Dietary fiber components, microstructure, and texture of date fruits (*Phoenix dactylifera*, L.)

**DOI:** 10.1038/s41598-020-78713-4

**Published:** 2020-12-10

**Authors:** Afaf Kamal-Eldin, Navomy George, Bhawna Sobti, Nouf AlRashidi, Sami Ghnimi, Abdul Aziz Ali, Annica A. M. Andersson, Roger Andersson, Asha Antony, Fathalla Hamed

**Affiliations:** 1 Department of Food, Nutrition, and Health, College of Food and Agriculture, United Arab Emirates University, Al Ain, 15551 UAE; 2 University of Lyon, Université Claude Bernard Lyon 1, CNRS, LAGEPP UMR 5007, 43 Bd 11 Novembre 1918, 69622 Villeurbanne, France; 3 Department of Economics and Statistics, Linnaeus University, Växjö, Sweden; 4 Department of Molecular Sciences, BioCentre, Swedish University of Agricultural Sciences, P.O Box 7015, 75007 Uppsala, SE Sweden; 5 Department of Veterinary Medicine, College of Food and Agriculture, United Arab Emirates University, Al Ain, UAE; 6 Department of Physics, College of Science, United Arab Emirates University, Al Ain, UAE

**Keywords:** Plant sciences, Structural biology

## Abstract

Date fruits vary widely in the hardness of their edible parts and they are classified accordingly into soft, semi-dry, and dry varieties. Fruit texture, a significant parameter in determining consumer acceptance, is related to the tissue structure and chemical composition of the fruit, mainly the ratio of sucrose to reducing sugars. This study aimed to understand the relationship between the chemical composition, microstructure, and texture profile of 10 major Emirati date fruits. The soluble sugars, glucose and fructose, represent ca 80 g/100 g of the fruits on the basis of dry weight (DW) while the dietary fiber contents varied 5.2–7.4 g/100 dg D.W. with lignin being the main determinant of the variability. The textures of the samples were studied using instrumental texture profile analysis. While no correlation was found between the soluble sugar and texture parameters in this study, the different fiber constituents correlated variably with the different parameters of date fruit texture. Lignin, arabinoxylan, galactomannan, and pectin were found to correlate significantly with fruit hardness and the related parameters, gumminess and chewiness. Both lignin and arabinoxylan correlated with resilience, and arabinoxylan exhibited a strong correlation with cohesiveness.

## Introduction

Texture is a useful quality to use for determining the consumer acceptance of fruits and how easy their processing will be. Bourne (2002) defined the textural properties of a food as “the group of physical characteristics that arise from the structural elements of the food that are sensed primarily by the feelings of touch, are related to deformation, disintegration and flow of food under force, and are measured objectively by functions of mass, time, and distance”^1^. Perceived texture is closely related to the structure and composition of foods at both microscopic and macroscopic levels. Thus, fruit texture is influenced by the chemical composition and cellular constitution of the fruit.

Date fruits at full maturity Tamr stage are primarily composed of sugars (60–80%) with the rest of the weight being moisture (10–30%), dietary fiber (5–12%), phenolic compounds (up to 4%), and other minor constituents on a fresh weight basis^2^. As they are variable in their moisture contents, date fruits are classified as soft (> 30% moisture), semi-dry (20–30% moisture), and dry (< 20% moisture) varieties on the basis of their moisture content. The types of sugar upon harvest, especially the sucrose to reducing sugars ratio, also plays an important role in classification of date fruits^3,4^. Most of the varieties grown in the United Arab Emirates and neighboring countries are soft and semi-dry dates. These varieties are mostly related despite some differences imposed by genetics, cultivation, and environmental factors^3,5^. In addition, differences in the content of soluble and insoluble dietary fibers contribute considerably to the variation between varieties^6,7^, and the varying levels of phenolic compounds contribute to the color and possibly texture of the fruit^8,9^. It has been suggested that texture of date fruit depends on anatomical cell wall structure, specifically skin cell size and shape of the underlying pericarp tissue layers^10,11^. In addition, it has been shown that the nanostructure of pectin, hemicellulose, and cellulose in cell walls affects the texture and firmness of pears^12^.

Microscopic analysis, such as light microscopy, can be employed to identify different tissue structures, such as phenolic compounds in the exocarp and mesocarp^9^. Moreover, scanning electron microscopy can be employed to study the structure and distribution of dietary fibers^13^. Another useful technique for analyzing food properties is texture profile analysis (TPA), which assesses food texture^14,15^. Through a double-bite compression test, TPA provides insight into how samples behave when they are chewed, and it quantifies multiple textural parameters, including hardness, cohesiveness, springiness, and resilience. In a previous study, we found substantial variability in the physical properties and texture profiles of fruits from 21 Emirati date varieties^16^. Understanding the texture profiles requires relating them to chemical composition, especially sugars and fibers, which are believed to influence texture, particularly hardness, elasticity, and stickiness.

This study aimed to understand the relationship between the chemical composition and microstructure of ten major Emirati date fruits and their texture profile. Fruit carbohydrates, including the soluble sugars glucose and fructose and dietary fiber components cellulose, hemicelluloses, pectin, and fructans, were determined and studied in terms of their distribution in different fruit tissues/cells and their correlation with texture parameters, including hardness, adhesiveness, cohesiveness, gumminess, chewiness, springiness, and resilience. The findings of the current study provide a better understanding of the sensory preference for different date fruits and can also be applied in date processing sector.

## Results

### The carbohydrate composition of Emirati date fruits

Fruits from ten Emirati date fruit varieties were used to investigate the relationship between their carbohydrate composition, microstructure, and texture. Table 1 presents the percentage of soluble sugars and dietary fiber components in the fruits on dry weight basis. These date fruits were dominated by the soluble sugars glucose and fructose that represented 38.2–44.7% and 36.8–40.1% of the total dry mass, respectively. The contents of total dietary fiber in these varieties varied 5.3–8.6% with the primary component being lignin (1.2–3.2%)^17^. A considerable variation was also observed in the other dietary fiber components like fructans (0.1–0.5%) and other neutral sugar from gas chromatography (GC) values of dietary fibers such as glucose (1.2–1.5%), arabinose (0.3%), xylose (0.7–1.1%), galactose (0.3–0.7%), mannose (0.2%), and uronic acids (0.8–1.1%) constituting cellulose/β-glucan (1.2–1.5%), arabinoxylan (1–1.4%), and galactomannan (0.5–0.9%). The order of varieties in terms of dietary fiber content was Neghal > Dabbas > Sagei > Shishi > Fardh > Reziz > Khalas > Boumann > Lulu red > Barhi, which was related to their hardness (see below).

**Table 1:** Contents of soluble sugars, total dietary fiber, and dietary fiber components in 10 United Arab Emirates date fruit varieties (g/100 g dry weight).

Variety	Soluble sugars		Total dietary fiber	Dietary fiber component					
	Glucose	Fructose		Fructans	Cellulose + β-Glucan	Arabinoxylan	Galactomannan	Lignin	Pectin
Barhi	44.7 ± 3.1^b^	38.3 ± 1.7^a^	5.19 ± 0.23^e^	0.42 ± 0.03^a,b^	1.24 ± 0.04^b^	1.05 ± 0.04^b^	0.47 ± 0.02^c^	1.24 ± 0.09^e^	0.78 ± 0.03^c^
Boumann	39.0 ± 1.3^ab^	36.8 ± 0.3 ^a^	5.97 ± 0.25^d,e^	0.13 ± 0.01^c^	1.27 ± 0.07^a,b^	1.09 ± 0.12^b^	0.73 ± 0.04^b^	1.93 ± 0.10^c,d,e^	0.82 ± .08^b,c^
Dabbas	40.9 ± 2.2 ^ab^	37.9 ± 0.9 ^a^	7.44 ± 0.40^a,b^	0.24 ± 0.06^b,c^	1.33 ± 0.05^a,b^	1.15 ± 0.05^b^	0.81 ± 0.05^a,b^	2.94 ± 0.24^a,b^	0.97 ± 0.01^a,b,c^
Fardh	41.3 ± 1.9 ^ab^	38.9 ± 0.4 ^a^	6.59 ± 0.25^b,c,d^	0.28 ± 0.04^a,b,c^	1.31 ± 0.11^a,b^	0.98 ± 0.10^b^	0.76 ± 0.07^b^	2.35 ± 0.14^b,c^	0.92 ± 0.08^a,b,c^
Khalas	41.7 ± 0.9 ^ab^	40.1 ± 0.5 ^a^	6.06 ± 0.74^c,d,e^	0.36 ± 0.08^a,b,c^	1.46 ± 0.23^a,b^	1.11 ± 0.16^b^	0.56 ± 0.05^c^	1.64 ± 0.27^c,d,e^	0.93 ± 0.16^a,b,c^
Lulu red	42.1 ± 0.6 ^ab^	39.8 ± 0.9 ^a^	5.81 ± 0.38^d,e^	0.35 ± 0.08^a,b,c^	1.31 ± 0.09^a,b^	1.02 ± 0.09^b^	0.76 ± 0.05^b^	1.54 ± 0.17^d,e^	0.83 ± 0.04^b,c^
Neghal	38.2 ± 2.4^a^	39.4 ± 1.6 ^a^	8.29 ± 0.23^a^	0.28 ± 0.09^a,b,c^	1.54 ± 0.02^a^	1.43 ± 0.04^a^	0.74 ± 0.01^b^	3.18 ± 0.32^a^	1.12 ± 0.01^a^
Reziz	39.4 ± 1.6 ^ab^	37.6 ± 2.1 ^a^	6.52 ± 0.45^b,c,d^	0.43 ± 0.19^a,b^	1.42 ± 0.09^a,b^	1.11 ± 0.05^b^	0.55 ± 0.02^c^	1.99 ± 0.32^c,d^	1.02 ± 0.11^a,b^
Sagei	41.6 ± 1.4 ^ab^	37.1 ± 0.7 ^a^	7.31 ± 0.67^a,b,c^	0.30 ± 0.05^a,b,c^	1.25 ± 0.07^a,b^	1.06 ± 0.09^b^	0.89 ± 0.04^a^	3.00 ± 0.44^a,b^	0.81 ± 0.02^c^
Shishi	41.3 ± 1.1 ^ab^	38.3 ± 0.6 ^a^	6.89 ± 0.39^b,c,d^	0.50 ± 0.06^a^	1.30 ± 0.07^a,b^	1.13 ± 0.02^b^	0.70 ± 0.02^b^	2.33 ± 0.23^b,c^	0.91 ± 0.03^a,b,c^
Range	38.2–44.7	36.8–40.1	5.2–7.4	0.13–0.50	1.24–1.54	0.98–1.43	0.47–0.89	1.24–3.18	0.81–1.12

The value for each variety is the average of three samples collected at different locations in the UAE. The mean values ± standard deviation with different superscript letters within each column are statistically different (p < 0.001).

### The microstructure of date fruits

**Figure 1:**
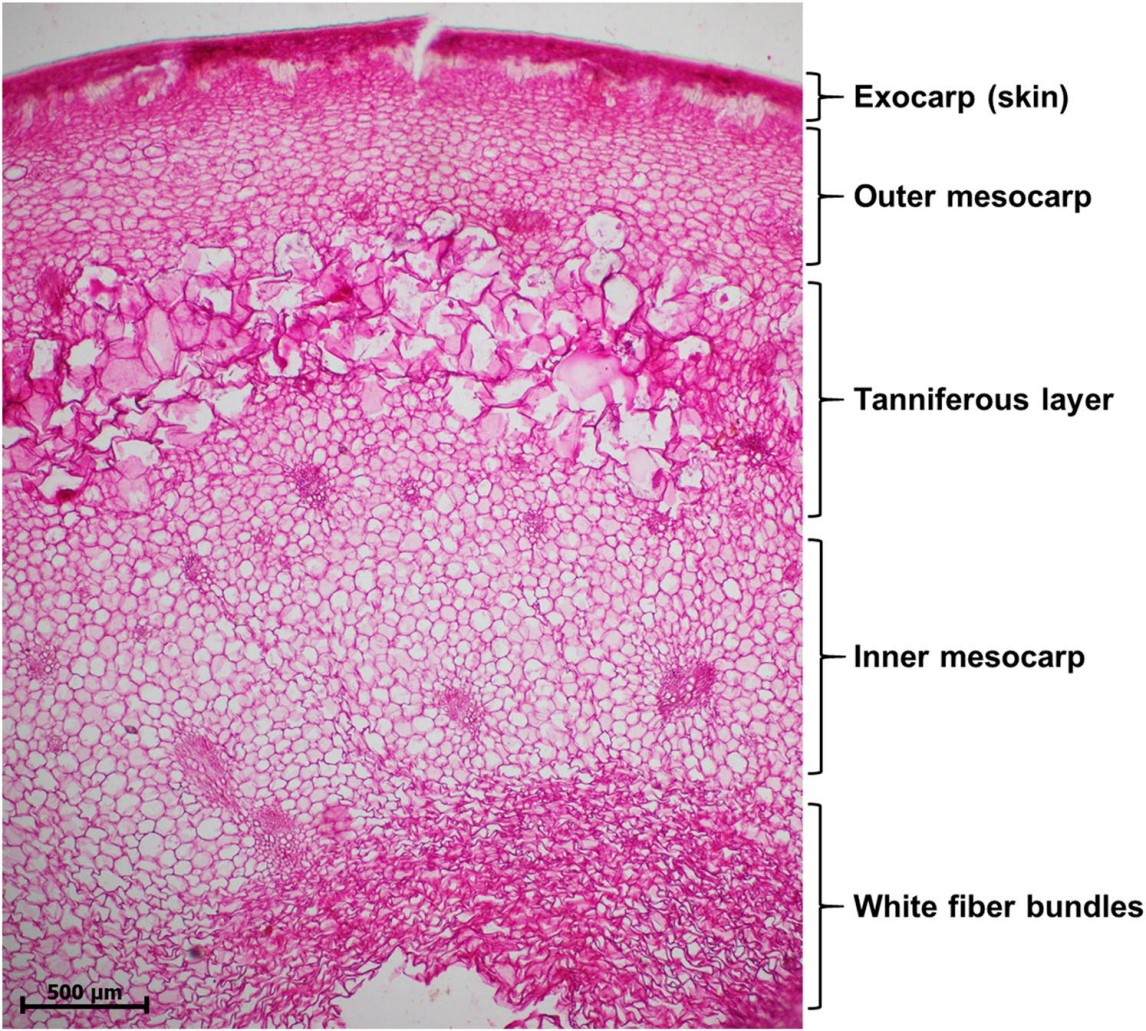
Light microscopy image of a date fruit specimen with a 5-µm thickness and viewed at a magnification of 4 × stained with Mayer’s hematoxylin, exhibiting the different tissues of the fruit: (1) the exocarp, (2) the outer mesocarp, (3) the tanniferous layer, (4) the inner mesocarp, and (5) the innermost white fiber bundles of the inner mesocarp.

**Figure 2:**
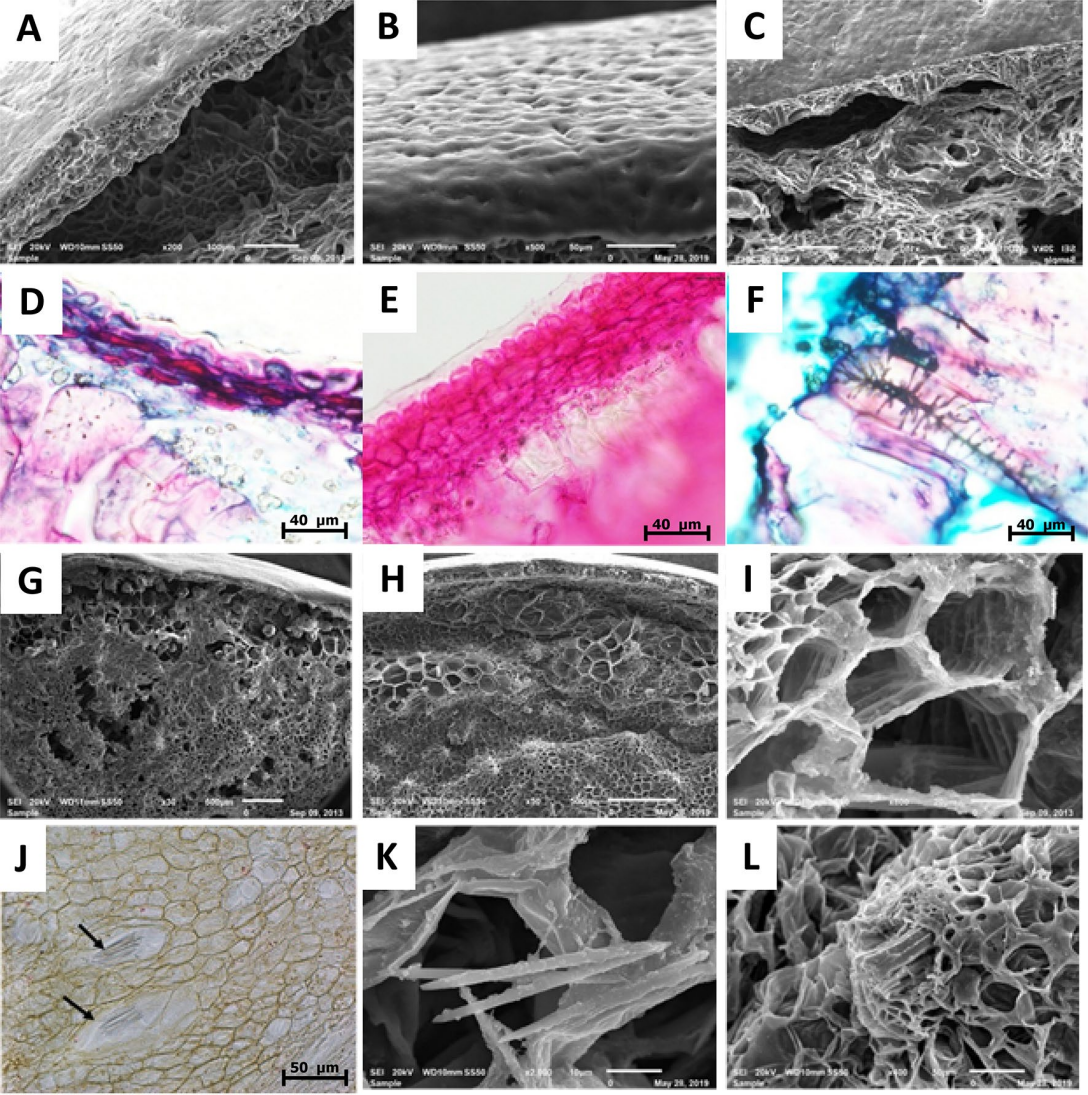
Micrographs of date fruit from a scanning electron microscope (**A**–**C**,**G**–**I**,**K**–**L**) and light microscope (**D**–**F**,**J**). (**A**) The cuticle, epidermis, hypodermis, and sclereid cells separating from the mesocarp; (**B**) the outer cuticle layer; (**C**) image focusing on the sclereid cells at the last cell layer of the skin; (**D**) the cuticle, epidermis, hypodermis, and sclereid cells (stained light pink); (**E**) enlarged image showing the single hypodermal cell layer, a two-cell hypodermal layer, and part of the sclereid layer surrounded by phytoliths; (**F**) an enlarged sclereid cell showing the secondary cell walls thickened by lignin; (**G**) the transition from the exocarp to the mesocarp and (**H**) the tanniferous layer; (**I**) enlarged parenchyma cells of variable sizes and shapes; (**J**) light microscopy images showing ideo blasts containing calcium oxalate needles in unstained specimen; (**K**) enlarged ideoblast showing calcium oxalate needles; and (**L**) an enlarged xylem vessels showing an emerging tracheid.

Figure 1 presents the microstructure of a date fruit (var. Sagei), at Kirmi stage (75 days after fruit set), stained with Mayer’s hematoxylin and observed via optical microscopy. The edible part of the date fruit, which is the flesh or pericarp, consisted of three distinguishable tissues: exocarp, mesocarp, and endocarp. The exocarp, or epicarp (Fig. 2A), consisted of five distinct layers: a non-cellular cuticle (Fig. 2B,C), an epidermal layer (Fig. 2D), a hypodermis (Fig. 2D), a skin parenchyma layer (Fig. 2E), and a layer of sclereid cells (Fig. 2F). A dense layer of epicuticular wax covered the surface of the fruit, except for the region close to the perianth. The single-cell epidermal layer was composed of small, elongated cells with cellulosic cell walls. Below the epidermis, we found the hypodermis, which was composed of one or two layers of tangentially elongated collenchyma cells. In some varieties, there was a layer of parenchyma cells, which are called the parenchyma of the skin. The hypodermis primarily consisted of collenchyma cells and was followed by highly lignified sclereid (or stone) cells. These sclereid cells, which represent the last layer of the skin, were elongated and radially oriented, with the long axis parallel to the fruit radius.

**Figure 3:**
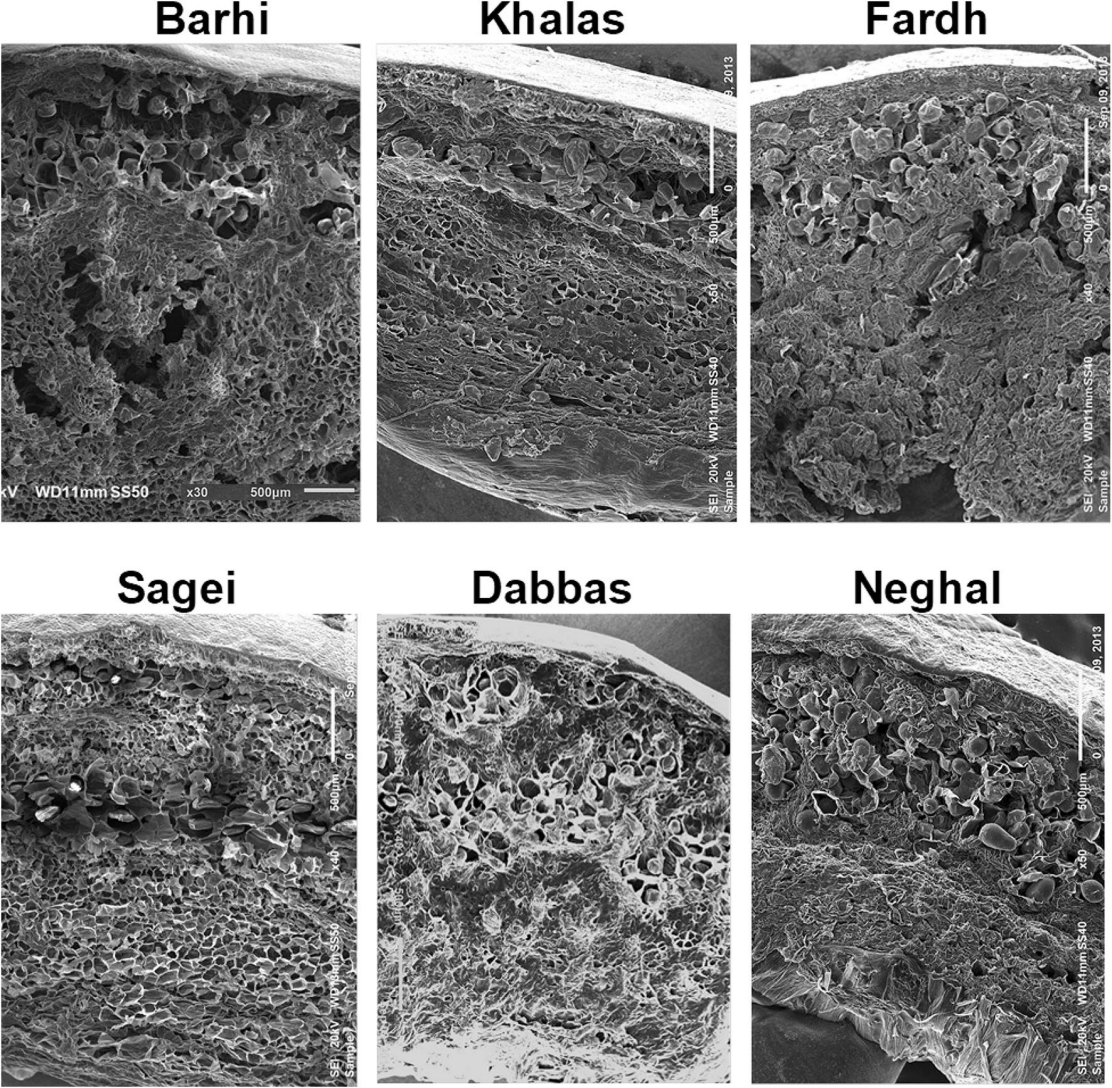
Variability of the microstructure of selected date varieties.

**Figure 4:**
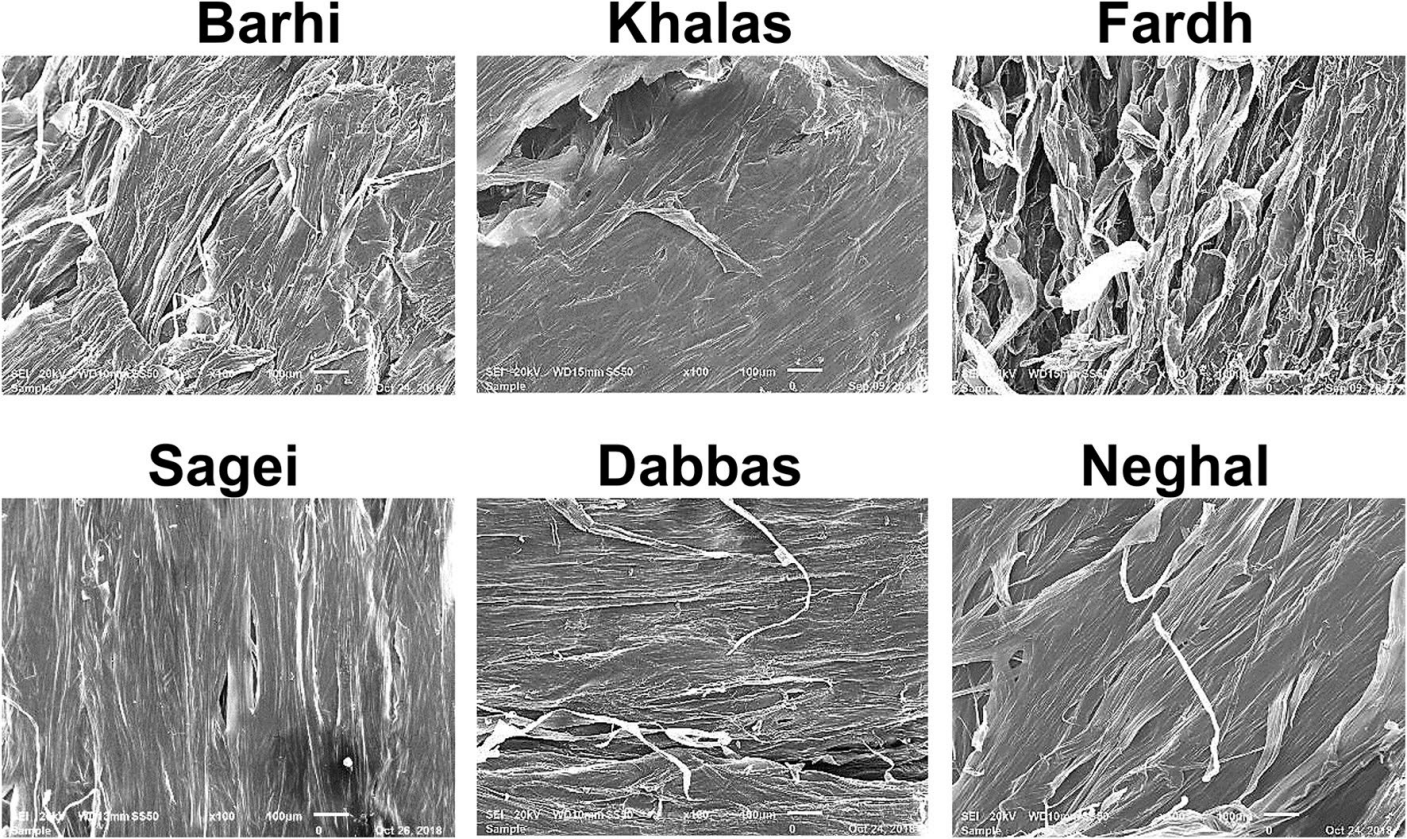
The internal white fiber bundles terminal to the mesocarp of selected date fruits.

The transition from the exocarp to the mesocarp varied between varieties, depending on the nature of the outer mesocarp and the tanniferous layer (Fig. 2G,H). The fruit mesocarp consisted of parenchyma cells organized into two morphologically distinct zones intermediated by 3–7 layers of tanniferous cells, which consist primarily of condensed polyphenols or tannins^18,19^. The inner mesocarp consisted of larger polyhedral parenchyma cells, which formed a spongy tissue and stored soluble sugars (Fig. 2H,I). At maturity, the mesocarp is not in direct contact with the seeds. In immature fruits, biomineralized calcium oxalate deposits occur as needlelike structures with pointed ends (Fig. 2J), which are called raphides. The specialized cell structures that contain them are called idioblasts (Fig. 2K)^20–22^. Vascular bundles (Fig. 2L), consisting of xylem and phloem elements, were scattered in various sizes all over the mesocarp with less abundance toward the interior. The variability in the microstructure in selected varieties is presented in Fig. 3. The portion in between the flesh and the seed coat was a distinct white part, the lower mesocarp, which was composed of fibrous bundles that are edible and devoid of sugars (Fig. 4). The thickness and nature of the white fibrous layers, which were adhered to the flesh of the fruit forming the lower mesocarp region, differed between the date fruit varieties.

### Instrumental texture profile analysis

**Figure 5:**
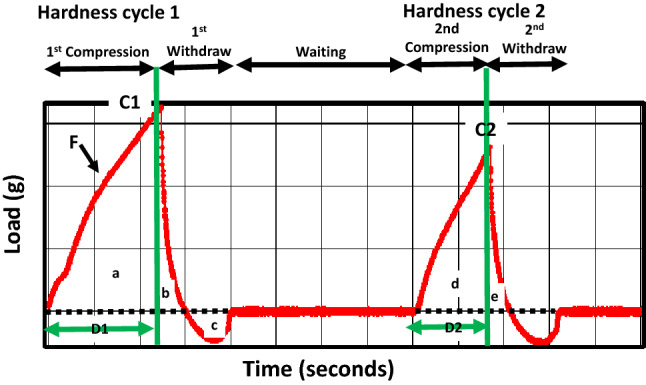
A typical texture profile of date fruits. The analysis is based on two-compression cycles mimicking bites with teeth. Hardness cycles 1 and 2 represent the forces required for the penetration of the food material during bite 1 and bite 2, and they are measured by the heights C1 and C2, respectively. Fracturability (F) is a measure of brittleness. Adhesiveness, a measure of stickiness to surfaces, is defined as the force necessary to pull the compressing probe out of the sample, and it is measured as the area above the curve for the first negative peak (c). Cohesiveness, or consistency, measures the internal bonds keeping the product intact and is calculated as (d + e)/(a + b). Springiness is the extent to which a deformed material returns to its initial condition and is calculated as D2/D1*100. Gumminess is the energy required to break a semisolid food into fragments, and it is calculated as the product of hardness and cohesiveness. Chewiness is the energy required to convert a solid food into a softer state suitable for consumption, and it is calculated as the product of hardness, cohesiveness, and springiness. Resilience describes the ability of the sample to return to its original form after being compressed, and it is calculated as the area under the curve after the peak force is reached divided by the area under the curve before the peak force is reached, i.e., b/a.

**Figure 6:**
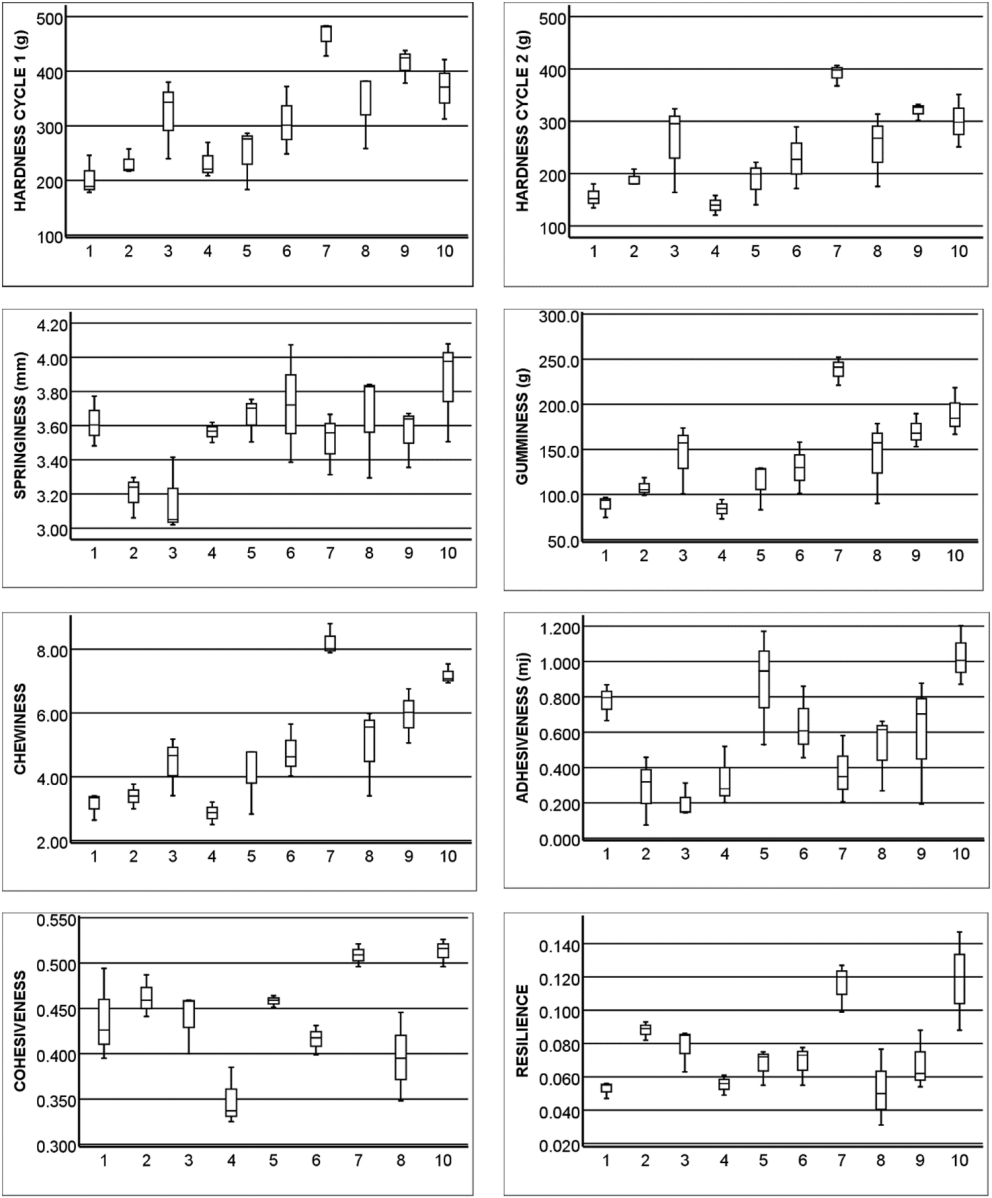
The variability of the texture parameters in the 10 studied date varieties: (1) Barhi, (2) Boumann, (3) Dabbas, (4) Fardh, (5) Khalas, (6) Lulu red, (7) Neghal, (8) Reziz, (9) Sagei, and (10) Shishi.

The instrumental TPA attributes were determined from the force–time curves presented and explained in Fig. 5. The ten date varieties studied here differed in their textural properties (Fig. 6). For example, Barhi and Lulu were found to be soft varieties, Neghal and Dabbas were found to be hard to semi-hard in texture, and Khalas and Fardh fell in between these. Table 2 presents the Pearson correlation coefficients between texture parameters, soluble sugars, and dietary fiber components. No correlation was found between the soluble sugars and texture parameters. Lignin and total dietary fiber were highly correlated with hardness, gumminess, chewiness, and resilience, whereas all other correlations were moderate. There were correlations between arabinoxylan and hardness, gumminess, chewiness, cohesiveness, and resilience; between galactomannan and hardness; between fructans and springiness and adhesiveness; and between cellulose + β-glucan and gumminess. In addition, we found no correlation between the silica content in the date fruits studied here and their texture properties (results not shown).

**Table 2:** Pearson correlation coefficients (r) for the correlations between total fiber and fiber components and the textural parameters of the ten date fruit varieties studied.

Carbohydrate polymer (%)	Hardness cycle 1 (g)	Hardness cycle 2 (g)	Springiness (%)	Gumminess (g)	Chewiness (g)	Adhesiveness (mJ)	Cohesiveness	Resilience
Cellulose + β-glucan	NS	NS	NS	0.364*	NS	NS	NS	NS
Arabinoxylan	0.484**	0.558**	NS	0.624**	0.585**	NS	0.623**	0.531**
Galactomannan	0.433*	0.402*	NS	NS	NS	NS	NS	NS
Pectin	0.407*	0.411*	NS	0.477**	0.446*	NS	NS	NS
Klason lignin	0.651**	0.635**	NS	0.600**	0.523**	NS	NS	0.363*
Fructans	NS	NS	0.590**	NS	NS	0.611**	NS	NS
Total dietary fiber	0.704**	0.692**	NS	0.692**	0.635**	NS	NS	0.420*

The correlations are significant at **p-value < 0.01, *p-value < 0.05, and *NS* non-significant.

## Discussion

The date fruits studied here were previously found to vary in their physico-chemical and textural properties^16^. In this study, we show that there is considerable cellular and tissue structural variation in date fruits (Fig. 3). This structural variation in date fruits is mainly governed by the concentrations of the different fiber constituents and sugar types (sucrose *versus* reducing sugars). As the varieties studied here did not differ significantly in their contents of reducing sugars (Table 1), the main differences observed can be attributed to the fiber constituents. This is evidenced by the correlations between the fiber components and the textural properties of the fruits (Table 2).

The structure of the mature pericarp and the mode of its development in the date monospermous berry have been described previously^26–29^. A single-cell thin membrane, which constitutes a papery endocarp, is the innermost layer of the pericarp that surrounds the seeds^16^. The single-cell very thin tissue of the endocarp has no significance from a textural or nutritional point of view. The exocarp and mesocarp tissues of the date fruit are composed of different types of cells, including collenchyma, sclerenchyma, and parenchyma cells, as well as xylem vessels and phloem cells. Plant cell walls are complicated supramolecular assemblies whose composition and molecular organization play major roles in cell protection, cell–cell adhesion, and intercellular exchanges^30,31^. Cell walls are commonly composed of polysaccharides and lignin, but they also include some proteins and phenolic compounds, particularly ferulic acid^32,33^. Primary cell walls are built around cellulose molecules that align to form microfibrils with crystalline and non-crystalline regions. The cellulose microfibrils are grouped into fibril aggregates, which a diameter of 10–25 nm, which are mounted by a matrix of hemicellulose and either pectin or lignin^34,35^. Hemicelluloses, which are represented in date fruits by arabinoxylan and galactomannan (Table 1), are short-chain, amorphous polysaccharides that bind on the surface of cellulose microfibrils^36,37^, whereas pectin cross-links the hemicellulose molecules on adjacent microfibrils^38,39^. Lignin is an amorphous complex phenolic polymer that is abundant in secondary cell walls where it contributes stiffness and strength^40^. Phenolic acids, particularly ferulic acid, form covalent bonds that cross-link lignin to the arabinoxylan^41,42^.

The fruit exocarp is primarily composed of epidermal, hypodermal, and sub-hypodermal layers (Fig. 2A–F). The hardness of date fruits is primarily due to the collenchyma and, especially, the sclerenchyma cells of the exocarp. Collenchyma cells are simple elongated cells that are extremely elastic, allowing the cells to expand with the growth of the fruit. The walls of collenchyma cells are largely hydrated cellulose, but small amounts of hemicellulose and pectin have also been reported^43,44^. Collenchyma cells have unequally thickened primary cell walls, especially when observed in cross-sectional views^45^. The different thickness patterns of the walls are a characteristic feature, which is formed during elongation. It was reported that collenchyma cell walls contains many of the same polysaccharide components found in parenchyma cell walls but the proportions and chemical species were distinctly different^46^. Sclereid cells are a reduced form of sclerenchyma cells, whose name is derived from *scleros*, which means hard, and they have highly thickened, lignified cellular walls. The sclerenchyma manifested as sclereid cells have very hard secondary cell walls that are thickened by lignin.

The parenchyma cells, the main cells of the mesocarp, have thin primary cell walls (Fig. 2I) that are composed of cellulose, arabinoxylan, galactomannan, and pectin. Depending on the variety and stage of maturation, the skin parenchyma may include aerenchymas, which are cells with large intercellular spaces, and chlorenchymas, which are cells containing chloroplasts, in immature fruits^47^. The storage parenchyma, with thin polyhedral primary cell walls, constitute the dominant cells in the fruit mesocarp. These cells are usually the main components of the soft tissues, which function as reserves of starch during the early stages of fruit development and sugars upon maturity. Parenchyma cells can have intercellular spaces and be spatially arranged, or they can be compressed and tightly arranged. The inner white cells in the bottom of the mesocarp form inner fiber bundles, which are special parenchyma cells that are void of soluble sugars that function to provide strength without rigidity^48^. In a previous study, we analyzed the inner fiber bundles of two date varieties, Fardh and Sagei, and found that their cellulose + β-glucan, arabinoxylan, galactomannan, and pectin contents were comparable to those of the total fruit, but they lacked lignin^17^. The absence of a correlation between sugars and texture observed in these varieties is explained by the restricted range of variability in the type of sugars and their contents, making the effect of sugars on texture more of a constant than a variable^49^. Previously, the hardness of date fruit was found to correlate with pectin, crude fiber, and moisture contents, adhesiveness was found to correlate with glucose content, and gumminess was found to correlate with fructose, glucose, and total sugar content^14^. Thus, the mesocarp tissue is expected to contribute variably to fruit texture, depending on its composition and structural characteristics. For example, we have demonstrated that the xylem vessels in the mesocarp are lined with helical coils, which are formed by the deposition of lignin and silica phytoliths in hollow vascular tracheids^50^.

The instrumental TPA provides information about the mechanical properties of the fruit (Figs. 5, 6 and Table 2), which are related to the sensory properties perceived by humans^23,24^. The values obtained for the texture properties in this study agree with previously reported texture property values for Saudi and Emirati varieties, including Barhi, Boumann, Khalas, Lulu, and Sagei, at the Tamr stage^10,25^. Hardness or firmness, defined as the necessary force to attain a given deformation, is the most commonly assessed parameter of date fruit texture^13^. Lignin, which is the primary determinant of fruit hardness, is highly predominant in the skin layers of the fruits and particularly in the sclereids. Previously, the exocarp layer of Fardh and Sagei was found to contain two to four times the levels of total fiber and its different components in the whole fruit^17^. In this study, arabinoxylan, galactomannan, and pectin were found to correlate significantly with fruit hardness and the related parameters of gumminess and chewiness, but cellulose + β-glucan was only correlated with gumminess. Both lignin and arabinoxylan were correlated with resilience, and arabinoxylan exhibited a strong correlation with cohesiveness. Arabinoxylan has a water holding capacity that affects the cohesiveness and gumminess of bread dough^51^. Only fructan was found to be positively correlated with springiness. However, it also had a significant positive correlation with adhesiveness. Springiness logically follows cohesiveness. The addition of *Agave tequilana* fructans to oat cookies resulted in the formation of crystalline aggregates with lowered water adsorption and increased springiness and cohesiveness^52,53^. Owing to their high sugar contents, date fruits may provide unique balance between adhesiveness and cohesiveness. Fruit hardness is influenced by the moisture content of the fruit, but this parameter was not investigated here as it requires equilibration of the different varieties to known moisture levels, as shown before^54^. These authors differentiated two characteristics in dried date fruits: an “elastic nature,” which is characterized by deformation in the first compression, indicating hardness, adhesiveness, and chewiness, and a “plastic nature”, which is related to the ability of the fruit to regain its original shape after the first compression, indicating cohesiveness, resilience, and springiness. Because differences in moisture contents between these varieties were not large, we excluded the effect of moisture and considered the soluble sugars and fiber contents on a dry weight basis. It has been found that adhesiveness/cohesiveness in sugary samples is a complex surface characteristic, which is related to stickiness and is not linearly related to moisture content^55^. Since fracturability was not observed in the soft date varieties during compression, the measured cohesiveness may not be an “ideal cohesiveness”^54^, which requires careful investigation in the future.

This is the first study demonstrating that the different fiber constituents correlate variably with different date fruit texture parameters (Table 2). The results will enable a better understanding of the sensory preference for different fruits^56^, and they can be used in various food processing applications^57^. However, our study has at least two limitations that need to be addressed in future research. The first relates to the varieties used in the study, which were all of the inverted sugar type. Genetic studies have identified two populations of date varieties, an Eastern pool consisting of accessions from Asia to Djibouti (including UAE) and a Western pool consisting of accessions from Africa. The Eastern varieties produce ∼ 77% soft dates and ∼ 7% dry dates compared with the North African varieties, which produce ∼ 52% soft dates and ∼ 31% dry dates^58,59^. As mentioned above, there is variability in the moisture content of date fruits upon harvest^3,4^, and the soft varieties primarily store sugars as inverted sugars, glucose and fructose, whereas the dry varieties have variable levels of sucrose. It has been shown that there is a difference between crystalline sucrose and non-crystalline inverted sugars, especially fructose, in their ability to adsorb moisture with the presence of inverted sugars promoting a non-crystalline state leading to a plastic condition^60^. The second limitation is the disregard of the contribution of phenolic compounds to the texture of date fruits. Date fruits contain high concentrations and a wide range of phenolic compounds, including polymeric tannins that might contribute to date fruit texture^8,9^. Inclusion of dry varieties in future research efforts will enrich the data and highlight the contribution of sucrose content/ratio as well as the textural attributes. On the other hand, the contribution of phenolic compounds seems to be small and it will be complicated by the difficulty in their complete analysis and classification. Yet, it might add precision through small modifications of the fiber effects presented in this paper.

## Materials and methods

### Date fruits

Three samples of ten different Emirati date fruits varieties at the mature (Tamr) stage, were received from Al Foah date factory (Al Saad, Abu Dhabi, UAE): Barhi, Boumann, Dabbas, Fardh, Khalas, Lulu red, Neghal, Reziz, Sagei, and Shishi.

### Analysis of fiber components and soluble sugars

Dietary fiber components were analyzed using the Uppsala method, as described previously^17,61^. For the analysis of soluble sugars, 1 g of date fruit samples were weighed in falcon tubes, homogenized using an Ultra-Turrax homogenizer, and extracted four times using 10 mL of 0.1% orthophosphoric acid and intermittent centrifugation at 4600 rpm for 15 min at 4 °C. The supernatants were pooled in 50 mL volumetric flasks, and the volume was adjusted. Then, the samples were filtered through a 0.45-μm filter membrane into vials and analyzed via high-performance liquid chromatography. Separations were conducted on a 300-mm-long μ-Bondapak NH_2_-column (Waters Corporation, Milford, Massachusetts, USA) with an i.d. of 3.9 mm and particle size of 10 mm using 83:17 (v/v) acetonitrile/water as the mobile phase at a flow rate of 1.5 mL/min. Peaks were detected using a diode array detector at 190 nm and quantified against authentic standards of glucose and fructose (Sigma-Aldrich Corporation, Darmstadt, Germany).

### Light microscopy

Hand-cut sections of immature dates were stained with Mayer’s hematoxylin (HiMedia, Kennett Square, Pennsylvania, USA). The commercially available pre-prepared stain was added to sections placed on glass slides and retained for 40–60 s. Excess stain was washed off using de-ionized water, a cover slip was added, and a microscope was used to observe the sections in aqueous media. Double staining with Safranin and Fast Green was also used to observe the tissues of mature date fruits. Before the observation of mature date fruits, frozen dates were thawed, cut into pieces of ~ 3 mm in length and breadth, and dipped in ethanol in a series of 40%, 60%, and 80% for 30 min each and finally kept in 80% ethanol overnight for dehydration and removal of sugars. The next morning, the pieces were washed again in 80% ethanol, followed by two washes in absolute ethanol and two washes for 30 min and 1 h in xylene. The pieces were then embedded in paraffin, and radial sections of the fruits were prepared using a rotary microtome. The sections were progressively rehydrated, stained, and then dehydrated before observing them (mounted in a synthetic resin-based mounting medium), according to the procedure explained by Ma et al*.*^62^.

### Scanning electron microscopy

Date fruit pieces of ~ 3 mm in length and breadth were freed from the sugars by soaking them in 80% ethanol five times for 10 min, and then, they were dehydrated by washing twice with acetone. Next, the dehydrated pieces were mounted on aluminum studs using silver paint as an adhesive and conductor. The pieces were then sputter-coated with gold before observations. The scanning electron microscopy images of the fruit sections were obtained using an analytical scanning electron microscope (Jeol Analytical Scanning Electron Microscope, JEOL JSM-6010PLUS/LA, Tokyo, Japan). Scanning was conducted in a low vacuum using a power of 20 kV, and the images were collected in secondary electron imaging mode.

### Instrumental texture profile analysis

Instrumental TPA attributes were measured using a computerized CT3 texture analyzer equipped with a 4.5-kg load cell (TA instruments, Middleboro, MA, USA), which generates plots of force (g) versus time (s). A 2-mm-diameter penetration probe was used to measure the textural profile of the date fruit samples in the two-compression cycle model. All experiments were conducted at 25 °C ± 2 °C). One pitted date was divided into two equal halves, one side was placed over the other, and the middle of the date halves was penetrated at a 5-mm target value at a compression rate of 1 mm/s. The location of the middle of the date halves was selected for penetration on the basis of previous trials. All measurements were performed in 15 replications. The texture parameters of hardness cycles 1 and 2, adhesiveness, cohesiveness, gumminess, chewiness, and resilience were calculated using the built-in software program.
